# Investigation of a brief online verbal learning task

**DOI:** 10.1002/alz70857_099989

**Published:** 2025-12-24

**Authors:** Hannah Marx, Claudia Jacova, Frank Robertson, Jacklyn Gehling, Steven Harris, Ravneet K. Dhaliwal

**Affiliations:** ^1^ Pacific University, Hillsboro, OR, USA

## Abstract

**Background:**

In recent years, a significant effort has been made toward digitizing neuropsychological measures in an effort to increase accessibility. Results of this research demonstrate that digitized versions, when conducted in a supervised setting, are comparable to traditional paper‐and‐pencil tests. The validity of unsupervised online tests is less well known. In the present study we examined how an unsupervised, self‐administered online version of a list learning task compares to traditional versions among a cognitively healthy, older adult sample.

**Method:**

A community‐based sample (*n* = 279) of English‐speaking older adults, aged 60‐96 (*M* = 73.8), with a mean education of 14.9 (range=10‐20), majority female (52%), without existing neurocognitive diagnoses, self‐administered a Qualtrics online survey from home, which included a digitized list learning task based on the Rey Auditory Verbal Learning Task (RAVLT). Participants were visually exposed to, and asked to recall, a novel list of 15 words over the course of three learning trials. Then they were presented with an interference list, and later asked to recall the initial list of words following a delay. Total correct words recalled from each trial were summed, and mean score differences were compared across gender, years of education, and age quartiles (Q2=68, Q3=74, Q4=80).

**Result:**

Participants recalled an average of 5.4 (SD=2.7), 8.8 (SD=3.1), 10.0 (SD=3.2), and 7.0 (SD=3.9) words on Trials 1‐3 and Delayed Recall respectively. Females recalled more words across all trials (all t tests *p* < .05). Neither education or age were significantly correlated with performance; however, upon further inspection, those in the second quartile (ages 68‐73) recalled approximately one word more than the other age groups.

**Conclusion:**

Our results generally align well with published AVLT norms. They are also consistent with existing research suggesting that females perform better on verbal learning tasks. However, the absence of a relationship between performance and age or education suggests that the utilization of an unsupervised, digital verbal learning task must be approached with caution.

